# On the suitability of an allometric proxy for nondestructive estimation of average leaf dry weight in eelgrass shoots I: sensitivity analysis and examination of the influences of data quality, analysis method, and sample size on precision

**DOI:** 10.1186/s12976-018-0076-y

**Published:** 2018-03-06

**Authors:** Héctor Echavarría-Heras, Cecilia Leal-Ramírez, Enrique Villa-Diharce, Nohe Cazarez-Castro

**Affiliations:** 1 Centro de Investigación Científica y de Estudios Superiores de Ensenada, Carretera Ensenada-Tijuana No. 3918, Zona Playitas, Código Postal 22860, Apdo. Postal 360 Ensenada, B.C. Mexico; 2 Centro de Investigación en Matemáticas, A.C. Jalisco s/n, Mineral Valenciana, Código Postal 36240 Guanajuato Gto, Mexico; 3 Instituto Tecnológico de Tijuana, Calzada Tecnológico S/N, Fracc. Tomas Aquino, Código Postal 22414 Tijuana, Baja California Mexico

**Keywords:** Eelgrass conservation, Nondestructive estimation, Allometric projection suitability, methodological influences, Sensitivity analysis

## Abstract

**Background:**

The effects of current anthropogenic influences on eelgrass (*Zostera marina*) meadows are noticeable. Eelgrass ecological services grant important benefits for mankind. Preservation of eelgrass meadows include several transplantation methods. Evaluation of establishing success relies on the estimation of standing stock and productivity. Average leaf biomass in shoots is a fundamental component of standing stock. Existing methods of leaf biomass measurement are destructive and time consuming. These assessments could alter shoot density in developing transplants. Allometric methods offer convenient indirect assessments of individual leaf biomass. Aggregation of single leaf projections produce surrogates for average leaf biomass in shoots. Involved parameters are time invariant, then derived proxies yield simplified nondestructive approximations. On spite of time invariance local factors induce relative variability of parameter estimates. This influences accuracy of surrogates. And factors like analysis method, sample size and data quality also impact precision. Besides, scaling projections are sensitive to parameter fluctuation. Thus the suitability of the addressed allometric approximations requires clarification.

**Results:**

The considered proxies produced accurate indirect assessments of observed values. Only parameter estimates fitted from raw data using nonlinear regression, produced robust approximations. Data quality influenced sensitivity and sample size for an optimal precision.

**Conclusions:**

Allometric surrogates of average leaf biomass in eelgrass shoots offer convenient nondestructive assessments. But analysis method and sample size can influence accuracy in a direct manner. Standardized routines for data quality are crucial on granting cost-effectiveness of the method.

## Background

Based on published data for 17 ecosystem services in 16 biomes, Costanza et al. [1] estimated the value of ecosystem services at the level of the whole biosphere. They found a lower bound in the range of US$16–54 trillion (1012) per year, with an average of US$33 trillion per year. Marine systems produced near 63% of the annual value. Almost half derived from coastal ecosystems. Approximately 25% of this share related to algal beds and seagrasses. This contribution to human welfare is deem relevant. Thus maintaining the health of marine ecosystems is subject of scientific concerns. Currently, increasing anthropogenic pressures pose important threats. And global climate change could also threaten future viability of seagrass meadows [2, 3]. For instance, water quality and other local stressors promote unprecedented meadow loss [4–6]. This reduces mitigation of wave action [7] and filtration [8]. Diminishes food and shelter for a myriad of organisms [9–11]. Weakens nutrient cycling [12, 13], erosion abatement and shoreline stabilization [14–16]. Moderates support for detrital food web foundation [17]. And inhibits carbon sequestration [18, 19].

Eelgrass (*Zostera marina L*.) is a dominant along the coasts of both the North Pacific and North Atlantic [20]. This species supports communities known as among the richest and most diverse in sea life [21]. Contribution of organic materials for food webs in shallow environments [22] is noticeable. Indeed, eelgrass produced up to 64% of the whole primary production of an estuarine system [23]. Current deleterious effects of anthropogenic influences on eelgrass prompted special restoration strategies. Among remediation efforts replanting plays an important role [24–27]. Transplant success amounts to reinstatement of ecological functions of natural populations. Evaluation relies on monitoring standing stock and productivity of transplanted plants. Then comparing with assessments of a reference population, which usually settle nearby [28].

Combined biomass of leaves in shoots is an important component of standing stock. Assessments rely on the estimation of the biomass of individual leaves. This requires shoot removal followed by dry weight measurement procedures in the laboratory. Elimination of shoots could infringe damage to natural eelgrass populations [29]. And reduced shoot density makes these effects even more perceptible for transplanted plots. Allometric methods make it possible simplified-indirect estimations of eelgrass productivity and standing stock. Echavarría-Heras et al. [30] considered an allometric representation for eelgrass leaf biomass and related length. Agreeing with Solana et al. [31], the involved parameters are invariant within a given geographical region. Estimates and leaf length measurements grant nondestructive approximations of observed leaf biomass values. This way, leaf length measurements grant nondestructive approximations for observed leaf biomass values. Leaf growth rates estimation relies on successive measurements of leaf biomass. Then the allometric model in [30] entails nondestructive assessments of eelgrass productivity. But, invariance does not impede local factors to imply variability of parameter estimates. Besides, local influences other factors could explain numerical differences in parameter estimates. There are methodological influences that may render biased parameter estimates. Analysis method, sample size, and data quality can influence scaling results (e.g. [32, 33]). And, since scaling relationships are particularly sensitive to parametric uncertainties, Echavarría-Heras et al. [30] concluded that the actual precision of derived allometric surrogates requires clarification.

Here we deal with allometric surrogates for average leaf biomass in eelgrass shoots. These derive from the model ***w***(***t***) ***= βa***(***t***)^***α***^ for leaf biomass ***w***(***t***) and area ***a***(***t***) measured at time ***t***, and ***α*** and ***β*** parameters. Leaf area is more informative of eelgrass leaf biomass than corresponding length. Thus, the present scaling endures a boost in precision of parameter estimates by the model in [30]. This could increase the accuracy of derived surrogates for leaf biomass in shoots. Besides, eelgrass leaf area and length admit an isometric representation [34, 35]. Then, the time invariance found by Solana-Arellano et al. [31] also holds for parameter estimates of the present scaling. This by the way imbeds a nondestructive advantage to the present shoot-biomass substitutes. But, agreeing with Echavarría-Heras et al. [30], we must examine influences on precision of estimates for suitability of projections. Since, such an analysis was not produced before, we took here the try of filling that gap. Achieving the related goals, required the assemblage of an extensive data set. It comprises coupled measurements of eelgrass leaf biomasses and related areas. This is further called “raw data set”. A data cleaning procedure adapted from Echavarría-Heras et al. [30] removed inconsistent leaf biomass replicates from the raw data. Thereby forming what we call a “processed data set”. Differences in reproducibility strength allowed to assess data quality effects in precision. A similar procedure evaluated sample size effects. And a sensitivity analysis evaluated robustness of the projection method. This supports consistent, cost-effective allometric projections of observed values from raw data. But, this depends on nonlinear regression as an analysis method. Besides, sample size must be optimal. Data quality as expected improved reproducibility strength of the allometric projection method. But, this factor was more relevant in optimizing sample size. A detailed explanation of used procedures appears in the methods section. The results section is not only devoted to the presentation of our findings. It also examines the relative strengths of factors influencing the precision of proxies. A Discussion section emphasizes on the gains and the limitations of the method. Appendix 1 deals with the model selection problem. Appendix 2 is about data processing methodology. Appendix 3 presents the procedure for sensitivity assessment.

## Methods

The symbol *w*_*m*_(*t*) will stand for average leaf dry weight of shoots collected at sampling date *t*. An the average of these values over all sampling dates symbolized through 〈*w*_*m*_(*t*)〉. Formal representations of these variables appear in Appendix 3 (cf. Eq. (15) and Eq. (16)). The symbols *w*(*t*) and *a*(*t*) will one to one stand for biomass and area of an individual leaf collected at a sampling time *t*. We assume that these variables are linked through the scaling relationship

1 $$ w(t)=\beta a{(t)}^{\alpha }. $$

The present raw data come from a coastal lagoon located in San Quintin Bay, México [30]. This comprises 10,412 leaves and measured lengths [mm], widths [mm] and dry weights (g). The product of length times width provided estimations of leaf area [mm^2^] [36]. In what follows the symbol *n*_*ra*_ stands for number of leaves in raw data. Processed data results by applying direct and statistical data cleaning techniques. The direct hinges on the consistency of allometric models for eelgrass leaf biomass. Leaf length or area are allometric descriptors of eelgrass leaf biomass [30, 31, 34]. A model selection exploration corroborated a power function like trend assumption for the present data. Details appear in Appendix 1. Severe deviations, from the mean response curve, are inconsistent and must be removed. This took care of sets containing less than ten leaf dry weight replicates. The statistical procedure worked on sets with a larger number of replicates. It centers on properties of the median of a group of data. This is immune to sample size and also a robust estimator of scale. The adapted Median Absolute Deviation (MAD) data cleaning procedure [37] appears in Appendix 2. Processing data resulted in a number of *n*_*qa*_ = 6094 pairs of leaf dry weights and areas.

Parameter estimates $\widehat{\alpha}$ and $\widehat{\beta}$ and leaf area values yield allometric proxies ${w}_m\left(\widehat{\alpha},\widehat{\beta},t\right)$ for *w*_*m*_(*t*). (cf. Eq. (19)). The symbol $\left\langle {w}_m\left(\widehat{\alpha},\widehat{\beta},t\right)\right\rangle$ (cf. Eq. (20)) stands for the pertinent average over sampling dates. We use Lin’s Concordance Correlation Coefficient (CCC) [38] as an evaluation of reproducibility. This meant as the extent to which two connected variables fall on a line through the origin and with a slope of one. We represent this statistic by means of the symbol $\widehat{\rho}$. Agreement defined as poor whenever $\widehat{\rho}<0.90$, moderate for $0.90\le \hat{\rho}<0.95$, good for $0.95\le \hat{\rho}\le 0.99$ or excellent for $\widehat{\rho}$>0.99 [39]. Values of $\widehat{\rho}$ gave an evaluation of the strength of the ${w}_m\left(\widehat{\alpha},\widehat{\beta},t\right)$ devise to reproduce observed values.

In getting parameter estimates $\widehat{\alpha}$ and $\widehat{\beta}$ we relied on two procedures. The traditional analysis method of allometry and nonlinear regression. Assessing analysis method effects on reproducibility strength of ${w}_m\left(\widehat{\alpha},\widehat{\beta},t\right)$ depended on testing differences in $\widehat{\rho}$. The traditional approach involves a linear regression equation (cf. Eq. (4)). This obtained through logarithmic transformation of response and descriptor in Eq. (1). The nonlinear regression analysis method relied on maximum likelihood [40, 41]. This approach fitted the model of Eq. (1) in a direct way in the original arithmetical scale. The nonlinear fit allowed the consideration of homoscedasticity or heteroscedasticity (cf. Eqs. (5) and (6)). All the required fittings for both raw and processed data depended on the use of the R software.

We also fitted the model of Eq. (1) to samples of different sizes taken out from primary and processed data sets. Each sample of size *k*; with 100 ≤ *k* ≤ *n*_*ra*_ produced estimates $\widehat{\alpha}(k)$ for *α* and $\widehat{\beta}(k)$ for *β*, and resulting ${w}_m\left(\widehat{\alpha}(k),\widehat{\beta}(k),t\right)$ projections. The symbol $\widehat{\rho}(k)$ denotes the value of $\widehat{\rho}$ for a sample of size *k*. Differences in $\widehat{\rho}(k)$ allow exploring sample size influences in reproducibility.

Deviations *∆α*_*q*_ and *∆β*_*r*_ convey fluctuating values *α*_*q*_ = *α* + *∆α*_*q*_ and *β*_*r*_ = *β* + *∆β*_*r*_ for the parameters *α* and *β* one to one. The modulus of the vector of parametric changes (*∆α*_*q*_, *∆β*_*r*_) defines a tolerance range *θ*(*q*, *r*). And the value of *θ*(*q*, *r*) determined by the standard errors of parameter estimates denoted by mean of *θ*_*ste*_. A fixed value of *θ*(*q*, *r*) leads to four possible characterizations of the pair (*∆α*_*q*_, *∆β*_*r*_). Each one associates to a trajectory *w*_*m*_(*α*_*q*_, *β*_*r*_, *t*) shifting from a reference one *w*_*m*_(*α*, *β*, *t*). The symbol *δw*_*mθ*_(*α*_*q*_, *β*_*r*_, *t*) (cf. Eq. (42)) denotes deviations between reference and average of shifting trajectories at sampling dates. And the average of *δw*_*mθ*_(*α*_*q*_, *β*_*r*_, *t*) values taken over all sampling dates denoted through 〈*δw*_*mθ*_(*α*_*q*_, *β*_*r*_, *t*)〉 (cf. Eq. (43)). The absolute value of the ratio of 〈*δw*_*mθ*_(*α*_*q*_, *β*_*r*_, *t*)〉 to 〈*w*_*m*_(*α*, *β*, *t*)〉 defines a relative deviation index *ϑ*(*θ*). It measures sensitivity of 〈*w*_*m*_(*α*, *β*, *t*)〉 to fluctuations of tolerance *θ*(*q*, *r*) on *α* and *β*. Appendix 3 presents detailed formulae.

## Results

**Fig. 1:**
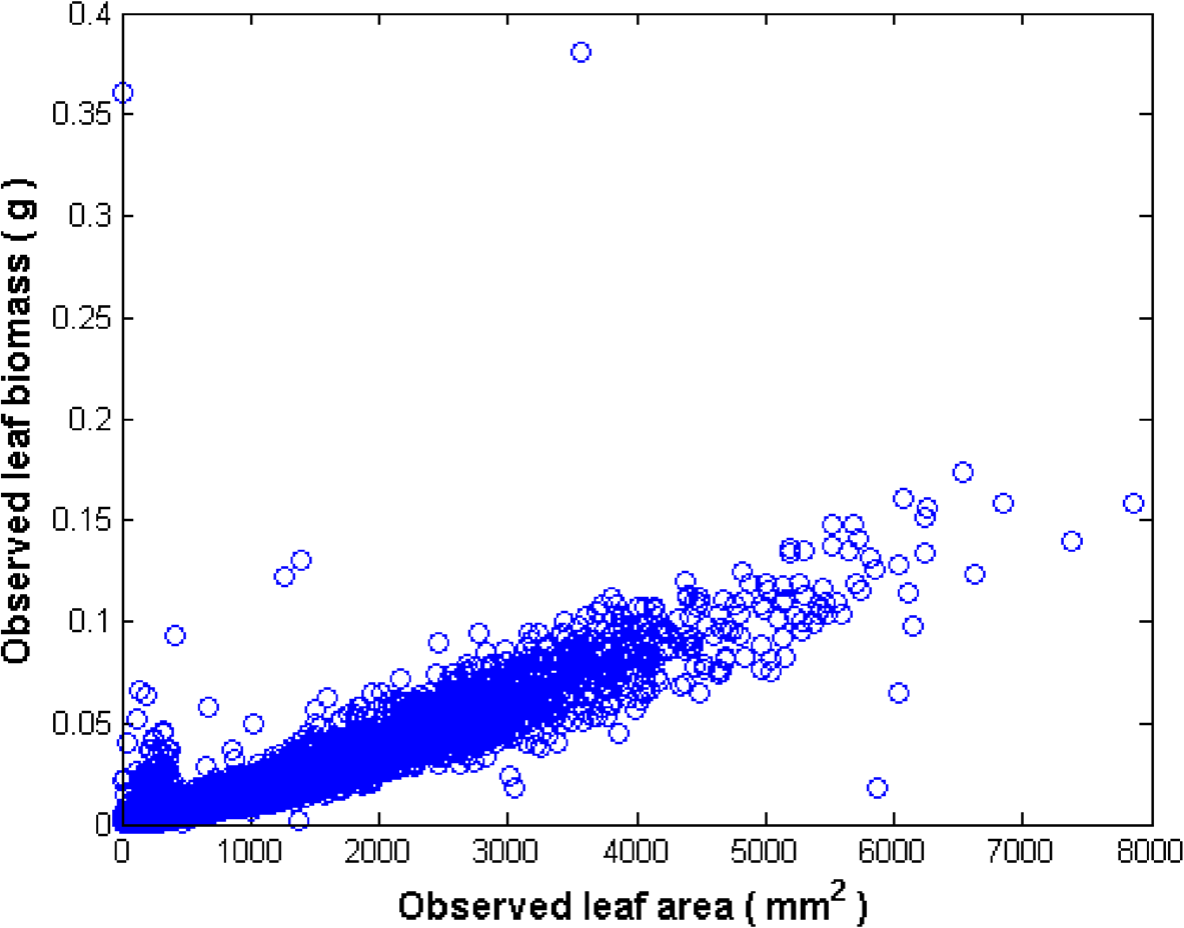
Distribution of eelgrass leaf dry weight and linked area values in raw data. Dispersion shows a masked power function-like trend. Deviations from this trend are more manifest for areas under 350 mm2 and also for those bigger than 5000 mm2. This suggests data quality effects on accuracy of allometric projections

Figure 1 shows the variation of leaf dry weight and area observed in the raw data. Smallest and largest leaf areas were 2 mm^2^ and 7868 mm^2^ respectively. Associated dry weights were 1 × 10^−5^ g and 0.1588 g one to one. The time average of mean leaf dry weight in shoots was 〈*w*_*m*_(*t*) 〉 = 0.01461g (cf. Eq. (16)). Each leaf area measurement associate to several replicates of leaf biomass. Number of replicates increased from a single association up to a largest value of 84. Dispersion masks a power function like trend. Contents of Appendix 1 corroborate this at formal level. And exploration of dispersion reveals severe deviations from the inherent power function-like trend. Inconsistencies are more visible for leaves with areas under 350 mm^2^ and also for those over 5000 mm^2^. This hints about the relevance of data quality.

**Fig. 2:**
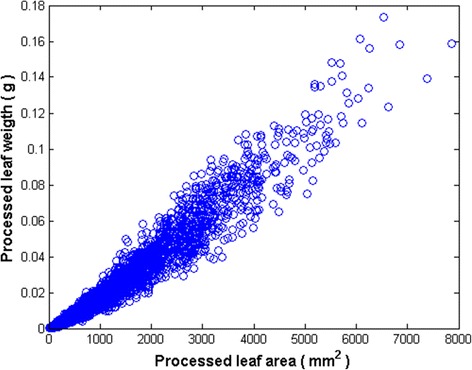
Plot of processed data. Distribution of eelgrass leaf dry weight and area values remaining after data quality control procedures. Although about 40% of the replicates in the raw data were found inconsistent and eliminated, this plot still shows significant residual variability around an expected power function like trend

Figure 2 exhibits the spreading of leaf dry weight after data quality control. About 40% of the replicates in the raw data were eliminated. A power function like trend appears more depicted than that showing on Fig. 1. But dispersion still shows significant deviations around the expected power function like trend. This suggests lack of standardized routines for data gathering. In this work the length times width proxy [36] approximated leaf area. Errors in estimation of area of older damaged leaves could explain uneven replicates. Faulty equipment, or incorrect recording could explicate inconsistencies for small leaves.

**Fig. 3:**
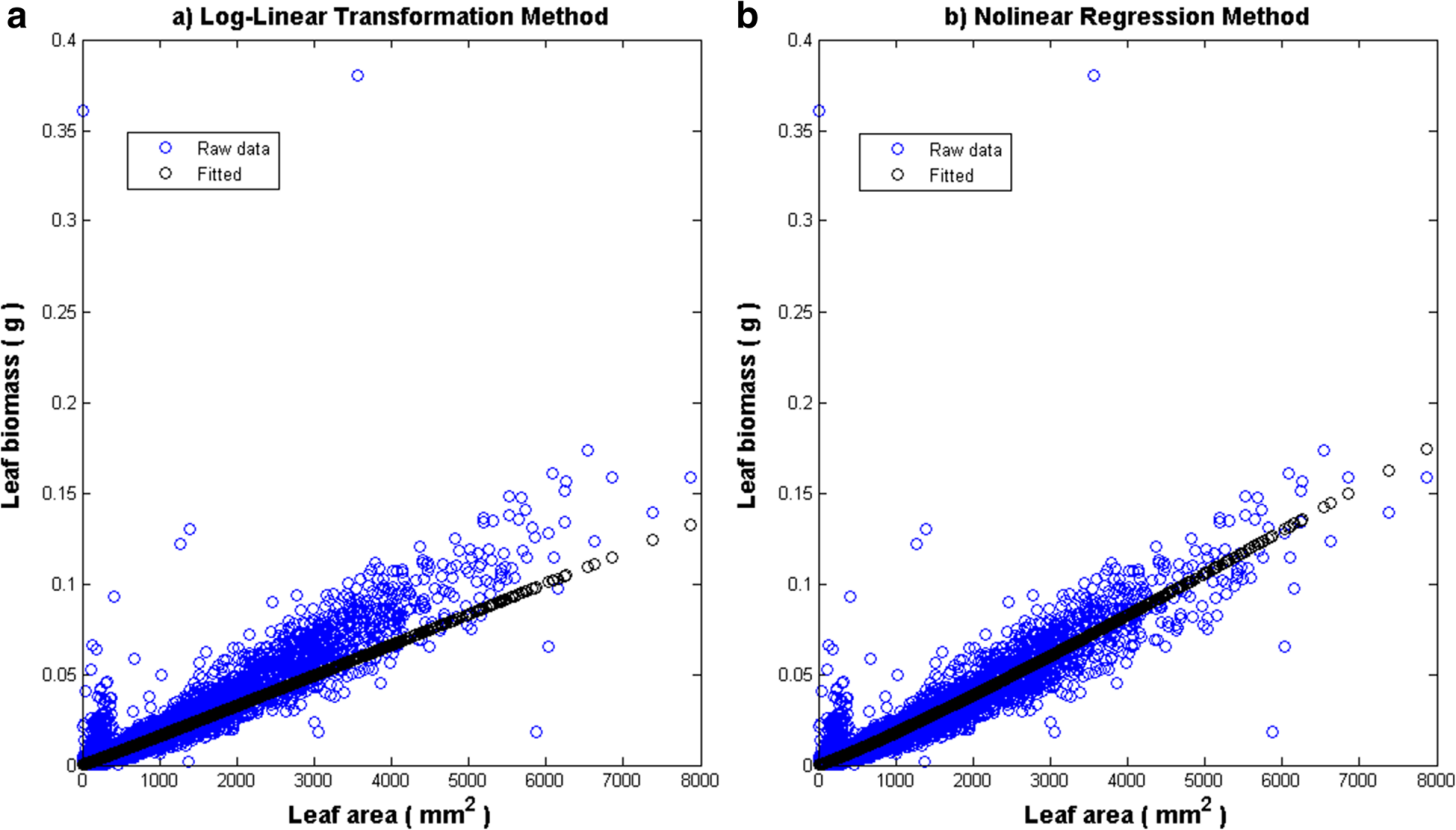
Fit of the model of Eq. (1) to raw data. Panel **a** Fitting results of the model of Eq. (1) by the log-linear transformation method. Distribution of replicates around the mean response curve shows a significant bias. This entails poor reproducibility ($\widehat{\uprho}$=0.8910) of leaf dry weight values. Panel **b** Shows fitting results for nonlinear regression and raw data. For this arrangement parameter estimates and Eq. (1) produced $\widehat{\uprho}$=0.9307. Thus, nonlinear regression stands a gain in reproducibility strength

**Fig. 4:**
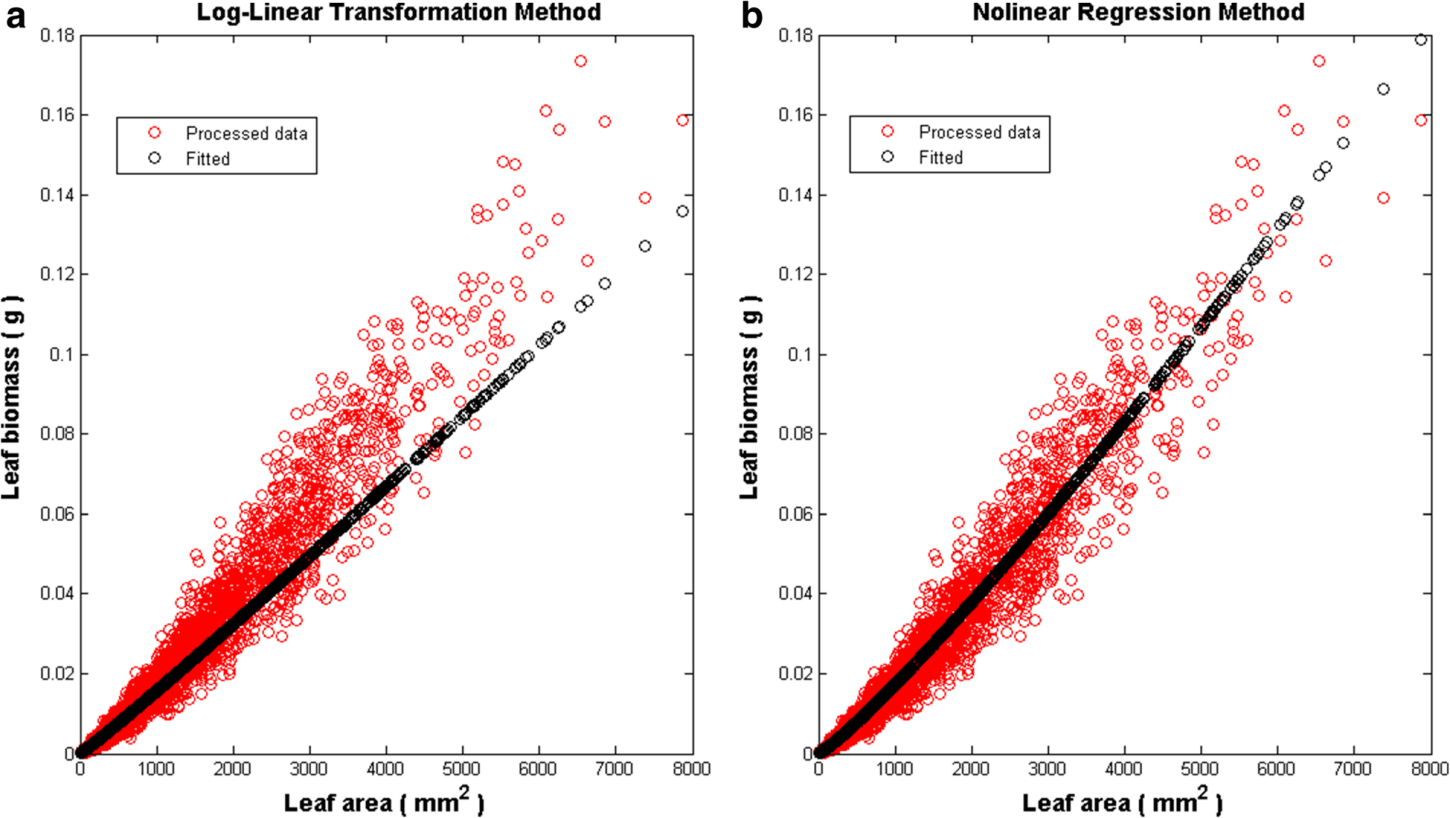
Fit of the model of Eq. (1) to processed data. Panel **a** Fitting results for model of Eq. (1) via traditional log-linear transformations. Though data processing improved goodness of fit, still a notorious bias remains. Panel **b** Fitting results by taking nonlinear regression as an analysis method. Shown spreading of replicates around the mean response curve is fair. Hence, $\widehat{\uprho}$=0.9777 entails suitable reproducibility of observed values via Eq. (1)

Table 1 gives estimates$\widehat{\alpha}$ and $\widehat{\beta}$for the parameters *α* and *β* and corresponding standard errors. Assuming heteroscedasticity in the model of Eqs. (5) and (6) did not affect estimates. Thus, presentation of results of nonlinear regression refers to the homoscedastic case of the model of Eqs. (5) and (6). Figure 3 displays mean response curves fitted using raw data. Figure 4 shows those associated to quality controlled data. Results for the log-linear transformation method included correction for bias of retransformation [42]. The smearing estimate of bias of Duan [43] provided the form of the correction factor.

**Table 1:** Parameter estimates $\widehat{\alpha}$ and $\widehat{\beta}$ associated standard errors ($ste\left(\widehat{\alpha}\right), ste\Big(\widehat{\beta}$)) found by fitting the model of Eq. (1). Nonlinear regression estimates associate to the homoscedastic case of the model of Eqs. (5) and (6) (see Appendix 1). Values of $\widehat{\rho}$ give an evaluation of reproducibility strength of the proxy of Eq. (1)

Analysis method Data	$\widehat{\beta}$	$ste\left(\widehat{\beta}\right)$	$\widehat{\alpha}$	$ste\left(\widehat{\alpha}\right)$	$\widehat{\rho}$
Log-linear Transformation Raw	1.3674x10^−5^	2.9355 × 10^− 7^	1.023	3.662 × 10^− 3^	0.8910
Nonlinear Regression Raw	8.718x10^−6^	3.530 × 10^−7^	1.104	5.101 × 10^−3^	0.9307
Log-linear Transformation Processed	1.142 x10^−5^	2.0831 × 10^−7^	1.046	3.035 × 10^−3^	0.9455
Nonlinear Regression Processed	6.956 x10^−6^	2.200 × 10^−7^	1.132	3.954 × 10^−3^	0.9777

For raw data the log-linear transformation method produced $\widehat{\rho}=0.8910$, entailing poor reproducibility. This explains a biased distribution of replicates around the mean response curve (Fig. 3a). Meanwhile, estimates acquired by nonlinear regression from raw data conveyed adequate reproducibility ($\widehat{\rho}=0.9307\Big)$. This explains a displayed fair distribution of projected leaf biomass values (Fig. 3b).

Estimates via log-linear transformation for processed data seemed enhance reproducibility ($\widehat{\rho}=0.9777$ ^=0.9455). But, Fig. 4a, reveals a bulk of inconsistent replicates for leaves areas under 5000 mm^2^. Notice that this subset of replicates distributes almost evenly around the mean response curve. Yet replicate spread for areas beyond 5000 mm^2^ shows significant bias (Fig. 4a). Meanwhile, nonlinear regression and processed data associate to $\widehat{\rho}=0.9777$. This corresponded to good reproducibility strength. Indeed, spread of replicates around the mean response is fair (Fig. 4b).

**Fig. 5:**
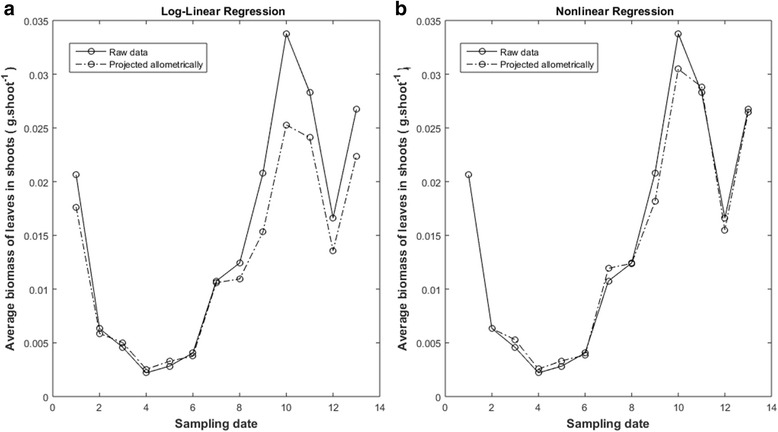
Effects of analysis method on reproducibility of *w*_*m*_(*α*,*β*,*t*) projections (raw data). Continuous lines display *w*_*m*_(*t*) averages of leaf dry weight in shoots. Dashed lines in panel **a** show *w*_*m*_(*α*,*β*,*t*) projections produced by log-linear transformation. Dashed lines in panel **b** display those projected via nonlinear regression. Nonlinear regression estimates support greater reproducibility of observed *w*_*m*_(*t*) values through *w*_*m*_(*α*,*β*,*t*) proxies (Table 2)

Estimates acquired from raw data via the traditional analysis method of allometry returned a value of $\widehat{\rho}=0.9285$ for ${w}_m\left(\hat{\alpha},\hat{\beta},t\right)$ projections (Table 2). This seems to correspond to moderate reproducibility. Yet, corresponding *rms* = 0.01265 was largest among analysis method- data set combinations (Table 2). Figure 3a shows a relative wider bias in spread around the mean response curve for larger leaves. This explains resulting inconsistencies in reproducibility of ${w}_m\left(\widehat{\alpha},\widehat{\beta},t\right)$ projections shown in Fig. 5a. Display reveals biased ${w}_m\left(\hat{\alpha},\hat{\beta},t\right)$ projections for near 50% of sampling dates. This, led to a smallest value of 0.8436 for the ratio of projected to observed averages.

**Table 2:** Reproducibility results for *w*_*m*_(*α*, *β*, *t*). Entries include, Lin’s concordance correlation coefficients $\left(\hat{\rho}\right)$, root mean square deviations (*rms*) and ratios of 〈*w*_*m*_(*α*, *β*, *t*)〉 to 〈*w*_*m*_(*t*)〉 averages

Analysis method Data	〈*w*_*m*_(*α*, *β*, *t*)〉/〈*w*_*m*_(*t*) 〉	$\widehat{\rho}$	*rms*
Log-linear Transformation Raw	0.8436	0.9285	0.01265
Nonlinear Regression Raw	0.9773	0.9915	0.00460
Log-linear Transformation Processed	0.8588	0.9489	0.01264
Nonlinear Regression Processed	0.9975	0.9976	0.00293

Instead, nonlinear regression and raw data produced a value of $\widehat{\rho}=0.9915.$ And root mean squared deviation attained a value of *rms* = 0.00460 (Table 2). This suggest a remarkable reproducibility strength for ${w}_m\left(\hat{\alpha},\hat{\beta},t\right)$ projections (Table 2). Correspondence between projected and observed values, shown in Fig. 5b. corroborates high agreement. Moreover, the ratio of projected to observed leaf dry weight averages attained an outstanding value of 0.9773 (Table 2).

**Fig. 6:**
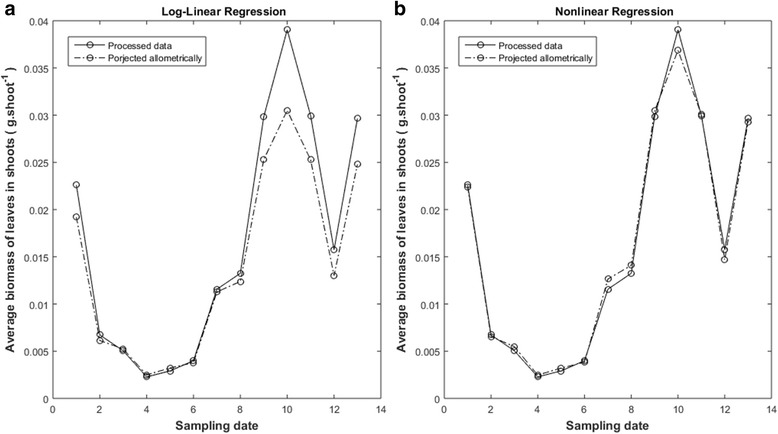
Effects of analysis method on reproducibility of *w*_*m*_ (*α*,*β*,*t*) projections (processed data). Continuous lines depict observed *w*_*m*_(*t*) averages. Dashed lines in panel **a**) are projections yield by log-linear analysis. Dashed lines in panel **b**) stand for projections linked to nonlinear regression

Processed data and log-linear transformation analysis produced $\hat{\rho}=0.9489$ for *w*_*m*_(*α*, *β*, *t*) projections. This figure is bigger than corresponding to raw data for this method. Nevertheless, lines in Fig. 6a show that this result does not correspond to a real gain in reproducibility. Besides data quality could not significantly reduce calculated root mean squared deviation (Table 2). Also, a value of 0.8588 for a ratio of projected to observed leaf dry weight averages is still low for suitable agreement (Table 2). Thus, regardless data quality, log-linear analysis failed to produce consistent *w*_*m*_(*α*, *β*, *t*) projections of *w*_*m*_(*t*) averages.

In turn, *w*_*m*_(*α*, *β*, *t*) projections made by nonlinear regression and processed data yield the highest value of $\hat{\rho}=0.9976$. (Table 2). And also the smallest root mean squared deviation among analysis method–data set combinations (Table 2). As shown by Fig. 6b this corresponds to a fairly good reproducibility strength. Additionally, data quality and nonlinear regression led to an outstanding value of 0.9975 for the ratio of projected 〈*w*_*m*_(*α*, *β*, *t*)〉 to observed 〈*w*_*m*_(*t*)〉 averages.

**Fig. 7:**
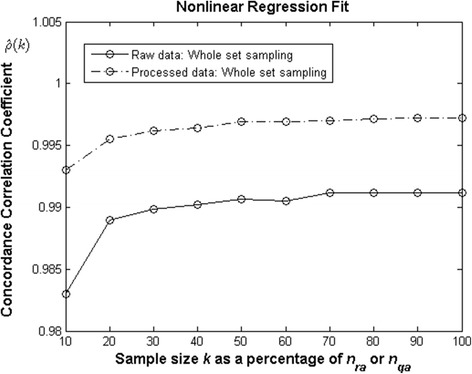
The effects of sample size on reproducibility of *w*(*α*,*β*,*t*). For raw data a sample of size *k* = 0.20n_*ra*_ (or near 2000 leaves) yields reasonable reproducibility ($\widehat{\uprho}$(*k*) = 0.9889). But, for quality controlled data similar reproducibility associates to only *k* = 0.10n_*rq*_ (about 1000 leaves). Larger sample would not induce a significant changes in the values of $\widehat{\uprho}$(*k*)

Results exhibit that log-linear transformations failed to produce consistent projections for observed *w*_*m*_(*t*) averages. In contraposition, nonlinear regression entailed parameter estimates of noteworthy reliability. Thus studying sample size effects on reproducibility addressed only this analysis method. Figure 7 exhibits variation of CCC values depending on sample size *k*. This is expressed as a percentage of the extent of data set ( *n*_*ra*_ = 10412 for raw) or (*n*_*qa*_ = 6094 for processed). For raw data, a sample sized *k* = 0.20*n*_*ra*_ endures reasonable reproducibility $\left(\widehat{\rho}(k)=0.9889\right)$**.** But samples beyond this threshold would not induce a significant change in reproducibility. Meanwhile, for the quality controlled data, the sample size threshold for excellent reproducibility was *k* = 0.10*n*_*rq*_. This leads to a high reproducibility strength of $\widehat{\rho}(k)=0.9929$**.** Thus, a sample 10% the size of processed data set produced excellent reproducibility. Again sample sizes beyond this threshold failed to increase $\widehat{\rho}(k)$ values.

**Fig. 8:**
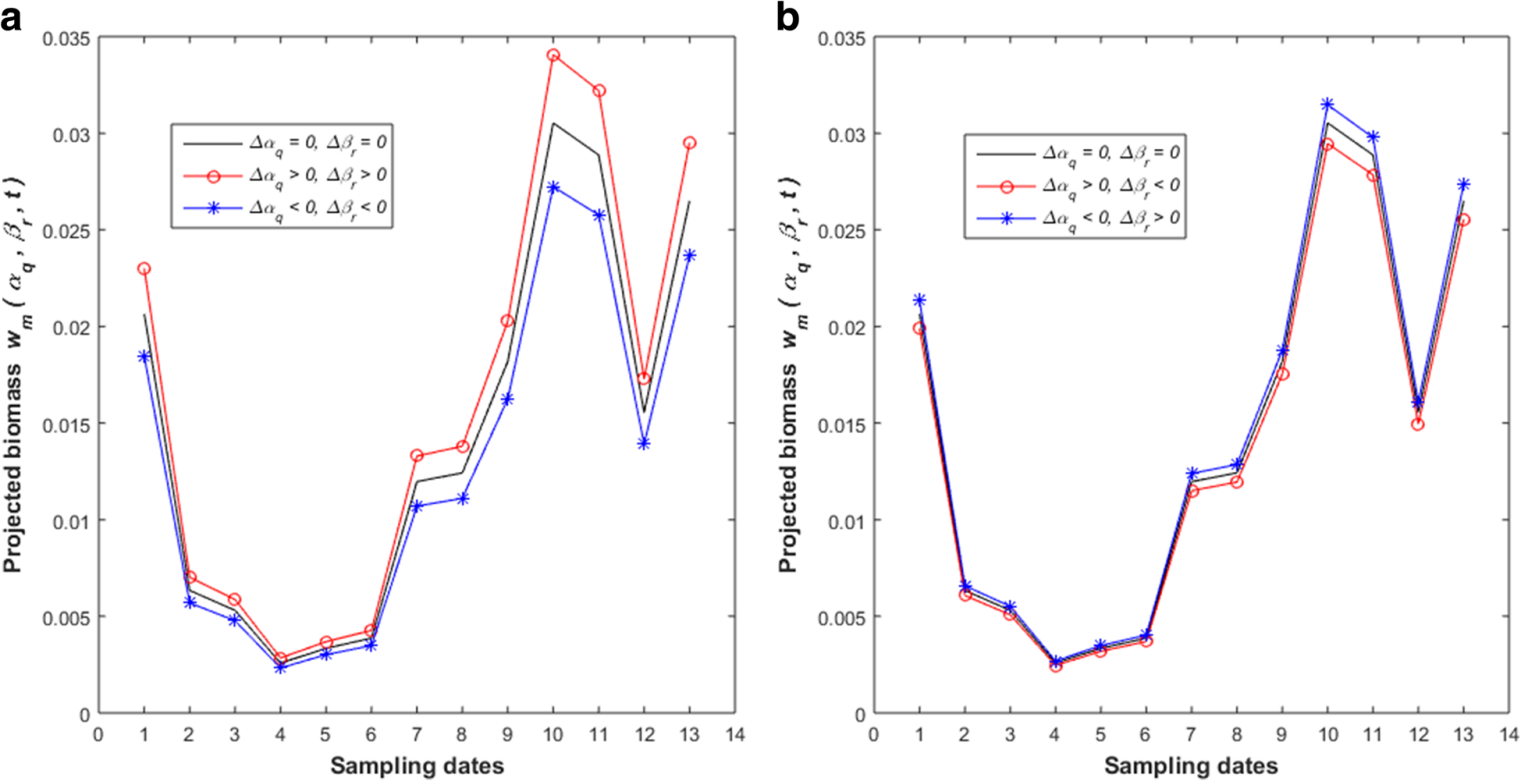
Examples of changing trajectories w_*m*_(*α*_*q*_, *β*_*r*_,*t*). Black lines a reference trajectory w_*m*_(*α,β,t*). This produced by raw data and nonlinear regression as an analysis method. For ∆*α*_*q*_. ∆*β*_*r*_ > 0, shifting trajectories w_*m*_ (*α*_*q*_, *β*_*r*_,*t*) overestimate or underestimate w_*m*_(*α,β,t*) projections (see red or blue lines in panel **a**)). The case ∆*α*_*q*_∙∆*β*_*r*_ < 0, leads to relative smaller deviations between w_*m*_(*α*_*q*_, *β*_*r*_,*t*) and w_*m*_(*α,β,t*) (see red and blue lines in panel **b**))

In Appendix 3 we consider variations *α*_*q*_ = *α* + *∆α*_*q*_ and *β*_*r*_ = *β* + *∆β*_*r*_. We found that shifting trajectories, *w*_*m*_(*α*_*q*_, *β*_*r*_, *t*) overestimated reference projections *w*_*m*_(*α*, *β*, *t*) whenever *∆α*_*q*_ > 0 and *∆β*_*r*_ > 0. In correspondence underestimation of *w*_*m*_(*α*, *β*, *t*) occurs for −*α* < *∆α*_*q*_ < 0 and −*β* < *∆β*_*r*_ < 0. Fig. 8 explains that for *∆α*_*q*_ ∙ *∆β*_*r*_ > 0, shifting trajectories overestimate (see red lines in panel a)) or underestimate the reference one (see blue lines in panel a)). We can also make certain that relatively smaller deviations between *w*_*m*_(*α*_*q*_, *β*_*r*_, *t*) and *w*_*m*_(*α*, *β*, *t*), values occur for the case, *∆α*_*q*_ ∙ *∆β*_*r*_ < 0, (see red and blue lines in panel b)).

The simulation code of Eqs. (39) through (44) explored the sensitivity of the *w*_*m*_(*α*, *β*, *t*) projection method, to numerical variation of parameters *α* and *β*. Available parameter estimates yield reference values for *α* and *β* (Table 2). Again, since nonlinear regression associates to highest reproducibility strength, for easier presentation, we only explain results using this analysis method.

**Fig. 9:**
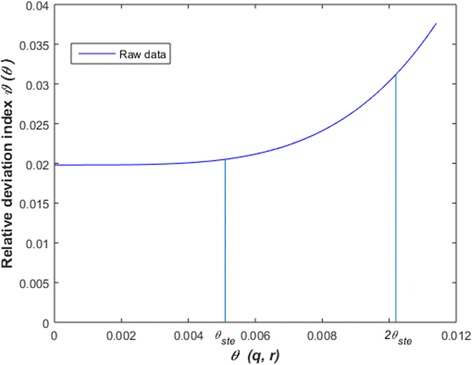
The variation of the relative deviation index ϑ(*θ*) (raw data). The standard errors of estimates acquired by nonlinear regression from raw data, produced *ϑ*(*θ*_*ste*_) = 0.0205. And for a range 0 ≤ *θ*(*q*,*r*) ≤ *θ*_*ste*_ the largest value of the absolute deviation between w_*m*_(*α*_*q*_, *β*_*r*_,*t*) and w_*m*_(*t*) is around 2% of 〈w_*m*_(*t)* 〉 (see Appendix 3 for details)

**Fig. 10:**
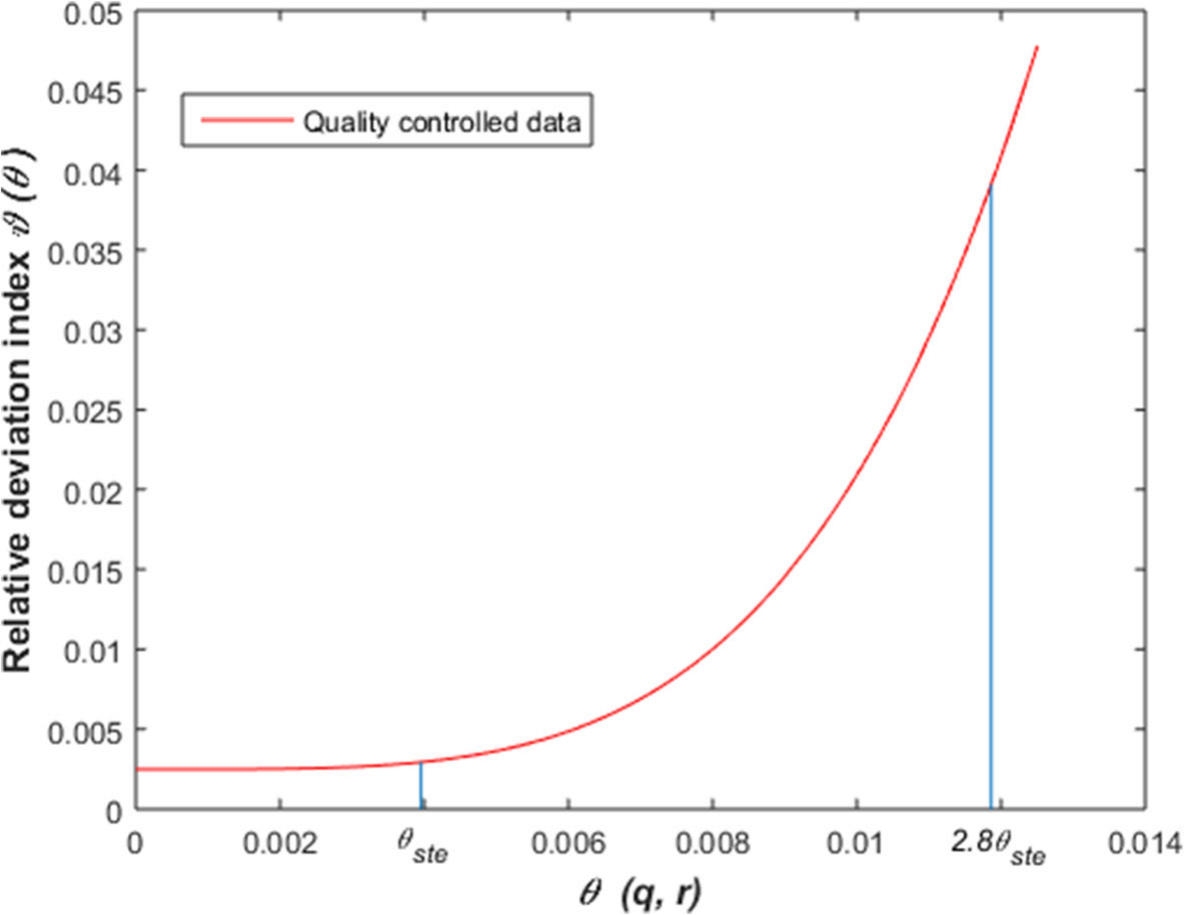
The variation of the relative deviation index *ϑ*(*θ*) (processed data). The standard errors of estimates acquired by nonlinear regression from processed data, produced *θ*_*ste*_ =3.954 × 10^− 3^). This set a range 0 ≤ *ϑ*(*θ*) ≤ 0.003. Thus, the largest absolute deviation between w_*m*_(*α*_*q*_, *β*_*r*_,*t*) and w_*m*_(*t*) amounts to about 0.3% of the 〈w_*m*_(*t*) 〉 average. Data quality control could be a factor improving accuracy of w_*m*_(*α,β,t*) projections

The variation of the absolute deviation index for raw data is shown in Fig. 9. A range 0 ≤ *θ*(*q*, *r*) ≤ *θ*_*ste*_, places the relative *ϑ*(*θ*) deviation index within the domain 0≤ *ϑ*(*θ*) ≤ 0.0205 (Table 3). Therefore, for a bound of *θ*(*q*, *r*) set by the standard errors of estimates largest absolute deviation between *w*_*m*_(*α*_*q*_, *β*_*r*_, *t*) and *w*_*m*_(*t*) amounts to about 2% of 〈*w*_*m*_(*t*) 〉. Moreover, a range 0 ≤ *θ*(*q*, *r*) ≤ 2*θ*_*ste*_ produces 0 ≤ *ϑ*(*θ*) ≤ 0.031. This leads to an equivalent 3% of 〈*w*_*m*_(*t*) 〉. Figure 10 displays the dynamics of *ϑ*(*θ*) depending on *θ*(*q*, *r*) for processed data. We have that *θ*(*q*, *r*) varying in a range of 0 ≤ *θ*(*q*, *r*) ≤ *θ*_*ste*_ implies 0 ≤ *ϑ*(*θ*) ≤ 0.003 (Table 3). This time largest absolute deviation was only 0.03% of 〈*w*_*m*_(*t*)〉. Comparing with results for raw data, we ascertained remarkable gain in precision of *w*_*m*_(*α*, *β*, *t*) projections. This exploration highlights on importance of data quality control as a procedure leading the consistent *w*_*m*_(*α*, *β*, *t*) projections. But, results show that the projection method is robust even when parameter estimates are obtained from raw data.

**Table 3:** Sensitivity of the *w*_*m*_(*α*, *β*, *t*) projections to changes in estimates of the parameters *α* and *β*. Included are calculated *θ*_*ste*_ values. This gives *θ*(*q*, *r*) as determined by the standard errors of estimates. We also present corresponding values of the relative deviation index *ϑ*(*θ*_*ste*_)

Analysis method Data	*θ*_*ste*_	*ϑ*(*θ*_*ste*_).
Log-Linear transformation Raw	3.662×10^−3^	0.1598
Nonlinear regression Raw	5.101 × 10^−3^	0.0205
Log-Linear transformation Processed	3.035×10^−3^	0.1419
Nonlinear regression Processed	3.954 × 10^−3^	0.003

## Discussion

Results of Solana-Arellano et al. [31] explain invariance of the allometric parameters *α* and *β* in Eq. (1). This suggest *w*_*m*_(*α*, *β*, *t*) proxies as possible nondestructive estimations of the average leaf dry weight in eelgrass shoots. These assessments are essential for monitoring the efficiency of transplanted eelgrass plots, fundamental in remediation aims. The present examination shows that the *w*_*m*_(*α*, *β*, *t*) proxies could in fact offer reliable and cost-effective assessments. This on condition that practitioners take in to account our guidelines. For instance, our results typify the extent on which accuracy of estimates of the parameters *α* and *β* influences the predictive power of the *w*_*m*_(*α*, *β*, *t*) projections. And, our findings clarify that there are methodological factors affecting the accuracy of estimates. Related influences could spread significant bias in approximations supported by the *w*_*m*_(*α*, *β*, *t*) device. Indeed, analysis method turned into a main factor inducing bias in parameter estimates of the model of Eq. (1). Moreover, only parameter estimates acquired by nonlinear regression yield consistency of the model of Eq. (1) (Table 1 and lines in Fig. 3b and Fig. 4b). And, only these estimates upheld conclusive predictive power of the *w*_*m*_(*α*, *β*, *t*) proxies (Table 2, as well as, lines in Fig. 5b and Fig. 6b). Our results also show that data quality could not improve the performance of *w*_*m*_(*α*, *β*, *t*) projections acquired via log-linear transformations. Without doubt, parameter estimates acquired from processed data by this method still led to significant bias in *w*_*m*_(*α*, *β*, *t*) projections (Fig. 6a). Meanwhile, data processing improved reproducibility of projections built for raw data using nonlinear regression (Table 2 and lines in Fig. 5b and Fig. 6b). Besides, relevance of data quality was also evident for optimizing sample size. Indeed, while for raw data, a sample of approximately 2000 leaves shows reasonable reproducibility, for the quality controlled data this threshold drops to near 1000. However, samples sized beyond these thresholds would not induce a noteworthy gain in reproducibility. This result on its own, ties to efficiency of the *w*_*m*_(*α*, *β*, *t*) projection method. Undoubtedly routines for leaf dry weight assessment are tedious and time consuming. So, reducing size of data set for parameter estimation increases cost-effectiveness of the *w*_*m*_(*α*, *β*, *t*) projection method.

Nonlinear regression estimation also showed advantages in sensitivity over the log-linear analysis counterpart. Estimates from raw data led to a largest absolute deviation between *w*_*m*_(*α*, *β*, *t*) and *w*_*m*_(*t*) values amounting only 3% of the average of *w*_*m*_(*t*) over sampling dates. And, for processed data, the fluctuation range for equivalent sensitivity widened to 2.8 times the range for standard errors of estimates. But, on spite of data quality relevance, sensitivity results for raw data reveal that the *w*_*m*_(*α*, *β*, *t*) projection method is robust relative to expected fluctuations in parameter estimates.

Our results show that both the accuracy and cost-effectiveness of projections can be enhanced by the addition of data quality control procedures. However, including data processing can become a weakness for the *w*_*m*_(*α*, *β*, *t*) projection method. Indeed, data cleaning procedures convey niceties that relate in a fundamental way to detection and rejection of inconsistent replicates. Also, compromising about which particular rejection edge should work, is hard to determine. Thus, the use of any data processing will endure a doubt, that the examiner selects an arrangement producing the most probable results [37]. In that order of ideas, when attempting to enhance the reproducibility power of *w*_*m*_(*α*, *β*, *t*) projections it is desirable to avoid depending in any form of data processing. For that aim, prior to data assembly, we must bear in mind standardized routines yielding accurate measurements for *w*(*t*) and *a*(*t*). This will favor direct identification of the model of Eq. (1) in a consistent way. It is of a fundamental importance to be aware, that errors in leaf dry weight or area assessment differentiate in terms of leaf size. Certainly, leaves produced anew normally present a complete and undamaged span. But, they normally yield very reduced dry weight values. Therefore, we can expect estimation errors imputable to the precision of the analytical scale for individual leaf dry weight assessments. To this, we may add errors in the reading and/or recording of the scale output. These issues could explain a perceptible accumulation of inconsistent replicates for leaves with areas between 2 mm^2^ and 350 mm^2^ (Fig. 1). And, even after data cleaning procedures, leaf dry weight replicate spread for leaves bigger than 2000 mm^2^ shows significant residual variability (Fig. 2). Likewise, as far as, bigger and older leaves is concerned, there are issues on dry weight estimation errors. These seem to relate to damage caused by exposure to environmental factors. The fact that we estimated leaf area by means of the product of related length and width could explain these effects. For complete undamaged eelgrass leaves, the use of a leaf times width proxy grants simplified and accurate estimations of leaf area [36].

But, this approach could deliver inaccurate estimations for long and damaged leaves. Actually, bigger leaves remain exposed during significant periods of time to environmental influences such as drag forces or herbivory. This could remove large amounts of leaf tissue while leaving length unaffected. Thus, causing overestimation of true leaf area when using a width and length product estimation. At the same time, lost portions of a leaf will produce a smaller dry weight than expected for an overestimated area. These effects will bring dry weight replicates that deviate from the power function–like trend associated to the model of Eq. (1). Estimation bias for the dry weights of smaller and longer leaves could explain the anomalous proliferation of inconsistencies (around 41 % ) found while applying the proposed data cleaning procedure to the present raw data. These effects will propagate uncertainty of parameter estimates of the model of Eq. (1), influencing accuracy of the *w*_*m*_(*α*, *β*, *t*) projections. Hence, for the sake of consistent reproducibility of observed values via *w*_*m*_(*α*, *β*, *t*) projections, we need to be aware of these effects. And as elaborated, a good starting point for granting consistency, is appropriate gathering of primary data for the identification of the model of Eq. (1). This will make subsequent data cleaning procedures unnecessary.

## Conclusion

This research show that precise estimates of allometric parameters in Eq. (1) grant accurate non-destructive projections of the average leaf dry weight in eelgrass shoots. These projections offer efficient appraisal of eelgrass restoration projects, thereby contributing to the conservation of this important seagrass species. Our findings support views in Hui and Jackson [32], Packard and Birchard [33] and Packard et al. [44], on the relevance of analysis method in scaling studies. Indeed, we found that for assuring suitability of the *w*_*m*_(*α*, *β*, *t*) proxies, the use of nonlinear regression is crucial. On spite of claims that the use of the traditional log-linear analysis method is a must in allometric examination [45], exploration of spread of present crude data reveals curvature [46]. This explaining failure of the traditional analysis method to produce unbiased results for the present data. Besides proxies supported by nonlinear regression and raw data, are robust.

Data cleaning could only marginally enhance the accuracy of proxies produced by nonlinear regression and raw data. But results underline a relevant influence of data quality in setting optimal sample size for a suitable precision of parameter estimates. Nevertheless, the use of data cleaning procedures leads to intricacies. They in a fundamental way relate to choosing thresholds for rejection of detected inconsistencies, which are often regarded as subjective. Thus, instead of using later data cleaning, data gathering should seek for suitability. Special care must be taken when processing bigger and older leaves. These are often damaged or even trimmed so that their dry weights do not conform to a true weight to area relationship. Irregularities in raw data may also associate to biased estimation of leaf length or width. Moreover, in a lesser way faulty equipment for leaf dry weight assessment, rounding off, or even incorrect data recording could as well contribute.

Taking advantage of a time invariance of the parameters in Eq. (1) the *w*_*m*_(*α*, *β*, *t*) device could offer to the eelgrass conservation practitioner highly consistent and truly nondestructive assessments of the average value of leaf dry weight in shoots. But the explained guidelines on analysis method, sample size and data appropriateness are mandatory for cost-effectiveness. Moreover, the present results suggest that the use of the *w*_*m*_(*α*, *β*, *t*) method could be extended to other seagrasses species with similar leaf architecture to eelgrass.

## Figures and Tables

**Fig. 11:**
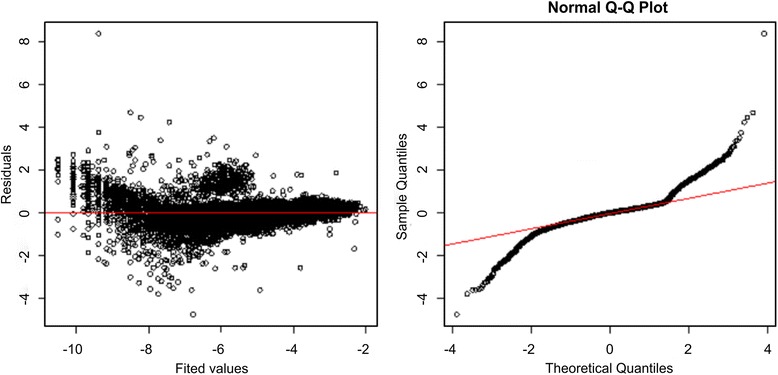
Residuals and normal probability plots for the fitting of the model of Eq. (4). This plot associates to a fit to raw data by means of the traditional analysis method of allometry. We observe a biased distribution of residuals around the zero line. Also, normal Q-Q plot shows heavier tails than expected for a normal distribution (left and right panels one to one)

**Fig. 12:**
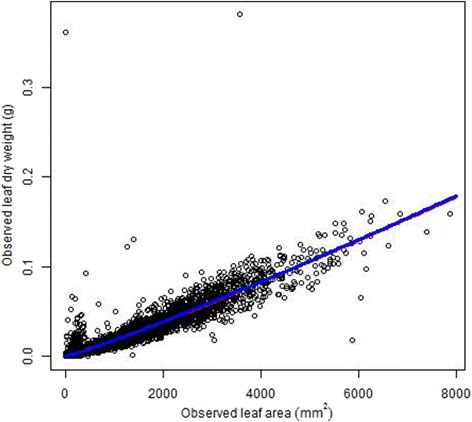
Data dispersion and allometric functions fitted through Eq. (5). This plot shows allometric function lines fitted by means of Eq. (5) and raw data. Homoscedasticity (blue lines) and heteroscedasticity (red lines) (cf. Eq. (6)). Notice that both lines overlap

**Fig. 13:**
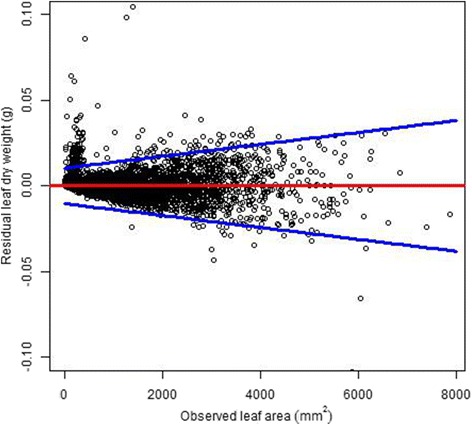
Scatter diagram of residuals of Eq. (5). This shows residuals against leaf area that associate to the fit of the regression model of Eqs. (5) and (6) allowing heteroscedasticity. Blue lines bound region 95% confidence intervals

**Fig. 14:**
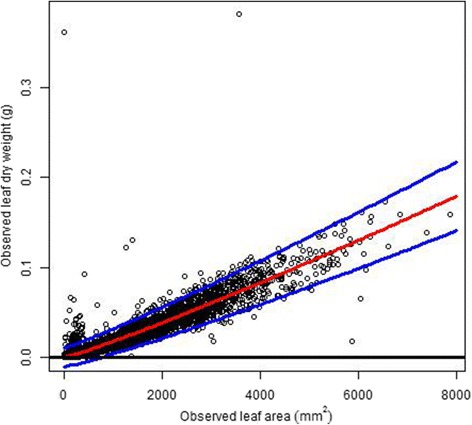
Dispersion around response curve fitted using Eq. (5) assuming heteroscedasticity. This displays leaf dry weight against leaf area in arithmetical scale and response curve (median) estimated by the heteroscedastic regression model of Eqs. (5) and (6) (red lines). Also shown is region comprising 95% of leaf dry weight values observed for a given value of leaf area

**Fig. 15:**
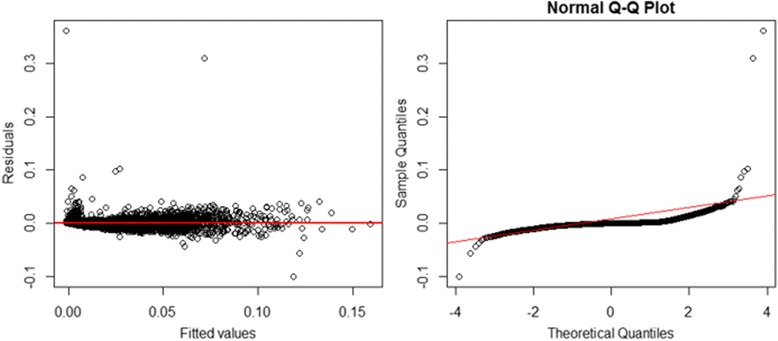
Residual and normal probability plots of fitting of Eq. (3). This shows the residual and normal probability plots resulting from the fitting of the linear regression model of Eq. (3) to raw data (left and right panels respectively)

**Fig. 16:**
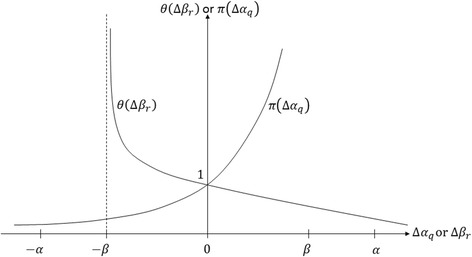
The variation of the auxiliary factors *φ*(∆*β*_*r*_)and *π*(∆*β*_*r*_). This plot presents the variation of the auxiliary factors *φ*(∆*β*_*r*_) and π(∆*β*_*r*_) defined by Eqs. (33) and (34) respectively. Both ∆*α*_*q *_and ∆*β*_*r*_r vary in the horizontal axis. Similarly, the corresponding variations of *π*(∆*β*_*r*_) and *φ*(∆*β*_*r*_) are projected in the vertical axis

## Appendix 1

### Model selection

This Appendix covers the selection problem of Eq. (1) as a model for the variation of eelgrass leaf dry weight in terms of related area. On first sight the spread in Fig. 1 may suggest that instead of clinging to the power function model of Eq. (1) we should explore fitting an alternative linear model. There are two possible ways to achieve a linear model fit for the present data. One by fitting an isometric model. This follows by setting *α* = 1 in Eq. (1) to yield a linear model with *w*-intercept through origin, that is,

2 $$ w(t)\kern0.5em =\kern0.5em \beta a(t) $$

A second choice is considering a standard linear equation with intercept different from zero, that is,

3 $$ w(t)=\beta a(t)+\delta, $$

where *β* is the slope and *δ* the intercept. But, by looking at the spread in Fig. 1, we realize that this model turns out to be inconsistent. This because for a vanishing leaf area we expect a vanishing dry weight. In any event, we fit the model of Eq. (3) as a device to analyze the suitability of the model of Eq. (2).

### Fitting of the model of Eq. (1) by means the traditional analysis method of allometry

For this fit we used raw data. Appling a logarithmic transformation on both sides of the Eq. (1), since we have available data pairs (*w*_*i*_, *a*_*i*_), we get

4 $$ \mathrm{In}\left({w}_i\right)=\mathrm{In}\kern0.28em \left(\beta \right)+\alpha \kern0.28em \mathrm{In}\kern0.28em \left({a}_i\right)+{\varepsilon}_i,i=1,2,\dots, n, $$

where the additive errors *ε*_1_, *ε*_2_, …, *ε*_*n*_ are independent and identically distributed, with distribution *N*(0, *σ*^2^). Fitting the model of Eq. (4) to data produced entries in Tables 4, 5 and 6.

**Table 4:** Residual statistics for the fitting of model of Eq. (4)

Minimum	1Q	Median	3Q	Maximum
−4.7535	−0.2642	0.0042	0.2151	8.3509

**Table 5:** Fitting results for the model of Eq. (4)

Parameters	Estimate	Std. Error	t value	Pr(>|t|)	Confidence Interval (95%)
*α*	1.022775	0.003662	279.3	<2e-16	(1.015597, 1.029953)
*lnβ*	−11.202199	0.021515	− 520.7	<2e-16	(− 11.24437, − 11.16003)

**Table 6:** Fitting tests for the model of Eq. (4)

Test	Value
Residual standard error	0.5723 on 10,410 degrees of freedom
Multiple R-squared	0.8823
Adjusted R-squared	0.8823
F-statistic	7.802e + 04 on 1 and 10,410 DF
*p*-value	< 2.2e-16

Residual and normal probability plots for the fit of the model of Eq. (4) appear in Fig. 11. We notice that a value *α* = 1.0 is not included in the confidence interval for the parameter *α*. This impairs Eq. (2) as a consistent model for the present raw data.

### Fitting of the model of Eq. (1) via non-linear regression

For the aim of fitting the model of Eq. (1) to raw data using nonlinear regression, we use the equation

5 $$ {w}_i=\beta {a_i}^{\alpha }+{\varepsilon}_i $$

the error term *ε*_*i*_ has a normal distribution with mean *μ* zero and standard deviation *σ*_*i*_. This chosen to contemplate heteroscedasticity or homoscedasticity. Moreover, *σ*_*i*_ depends on the predictor variable *a*_*i*_ through the model

6 $$ {\sigma}_i={\gamma}_0+{\gamma}_1{a}_i, $$

where *γ*_0_ and *γ*_1_ are parameters to be estimated. The case *γ*_1_ different from zero associates to heteroscedasticity. Whenever *γ*_1_ vanishes allows the consideration of homoscedasticity.

The response variable *w*_*i*_ resulting from Eq. (5) is normally distributed, with mean *μ*_*i*_ = *βa*_*i*_^*α*^ and standard deviation *σ*_*i*_ given by Eq. (6). Fitting this regression model to raw data relied on a likelihood approach, i.e., acquiring estimates for the parameters *β*, *α*, *γ*_0_ and *γ*_1_, which yield the largest value of the log likelihood function [40, 41]

7 $$ l\left(\beta, \alpha, {\gamma}_0,{\gamma}_1\right)=-\frac{n}{2}\log \left(2\pi \right)-{\sum}_1^n\log \left({\sigma}_i\right)-\frac{1}{2}{\sum}_1^n{\left(\frac{w_i-{\mu}_i}{\sigma_i}\right)}^2, $$

with *μ*_*i*_ = *βa*_*i*_^*α*^ and *σ*_*i*_ given by Eq. (6).

Estimates $\widehat{\beta}$and $\widehat{\alpha}$ are very similar for the homoscedastic and heteroscedastic cases of Eq. (5). Notice that a value *α* = 1.0 is not included in the confidence interval for the parameter *α*. Thus, this fit also excludes the model of Eq. (2) (isometric case).

Confidence intervals in Table 7 show a wide overlap region. It follows that parameter estimates do not differ in a significant way. This is also supported by the estimated CCC value ($\widehat{\rho}=0.9307$) that was the same for the heteroscedastic and homoscedastic cases. Likewise, plotting the response curves *w*_*i*_ = *βa*_*i*_^*α*^ on a dispersion diagram, reveals that curves estimated considering heteroscedasticity or homoscedasticity coincide (Fig. 12).

**Table 7:** Maximum likelihood estimates for parameters *β* and *α* in Eq. (5). This fit considers heteroscedasticity (*γ*_1_ ≠ 0) or homoscedasticity (*γ*_1_ = 0)

Parameters	Estimate	Std. Error	Confidence Interval (95%)
Homoscedastic model			
* α*	1.104	5.101 × 10^−3^	(1.094, 1.114)
* β*	8.718 × 10^−6^	3.530 × 10^−7^	(8.052 × 10^−6^, 9.433 × 10^−6^)
Heteroscedastic model			
* α*	1.107	6.116 × 10^−3^	(1.095, 1.119)
* β*	8.477 × 10^−6^	3.950 × 10^−7^	(7.703 × 10^−6^, 9.251 × 10^−6^)

Figure 13 shows the scatter diagram of dry weight residuals against leaf area. It also displays the region bounded by lines for 95% confidence intervals (blue lines). Figure 14 displays raw data and response curve (median) for the heteroscedastic model. It includes curves for region comprising 95% of leaf dry weight values (blue lines).

Thus, predictive strength from the homoscedastic or heteroscedastic regression models are similar. This because assumed distributions of the error term, in both models are symmetric. What makes a difference is the form capturing the uncertainty pattern.

### Fitting of the linear model of Eq. (3)

Since, raw data composes of pairs (*w*_*i*_, *a*_*i*_), in order to test the consistency of the model of Eq. (3), we consider the regression equation

8 $$ {w}_i=\beta\;{a}_i+\delta +{\varepsilon}_i, $$

where the additive errors *ε*_1_, *ε*_2_, …, *ε*_*n*_ are independent and identically distributed *N*(0, *σ*^2^). Fitting regression Eq. (8) to raw data produced the results in Table (8),Table (9) and Table (10).

**Table 8:** Residual statistics for the fit of Eq. (8)

Minimum	1Q	Median	3Q	Maximum
− 0.1004000	−0.0017230	0.000000	0.0010230	0.36210

**Table 9:** Fitting results for the model of Eq. (8)

Parameters	Estimate	Std. Error	t value	Pr(>|t|)	Confidence Interval (95%)
*β*	2.04e-005	7.838e-08	260.23	2e-16	(2.02e-005, 2.055e-005)
*δ*	−0.001158	8.954e-05	−12.93	2e-16	(−0.001158, − 0.0009825)

**Table 10:** Fitting test statistics for the model of Eq. (8)

Test	Value
Residual standard error	0.007279 on 10,410 degrees of freedom
Multiple R-squared	0.8668
Adjusted R-squared	0.8667
F-statistic	6.772e04 on 1 and 10,410 DF
*p*-value	2.2e-16

Residual and normal probability plot for this fit appear in Fig. 15. Looking at the confidence intervals in Table 7, we observe that a value *α* = 1 is not included. This implies rejection for the model of Eq. (2) (isometric case). Also, the intercept in Eq. (8) is negative, which is inconsistent. This is inconsistent with observations. Thus, we must reject the model of Eq. (3).

In summary, fitting results for the model of Eq. (1) excludes the value *α* = 1. Now, for *α* = 1, the model of Eq. (1) takes the form of a straight line through the origin (isometric model of Eq. (2)). In the other hand, for the fit of the linear model of Eq. (3) the intercept is negative. This means that this model could predict negative values of leaf dry weight. Thus, the model of Eq. (1) is the only one supporting a good selection criterion.

## Appendix 2

### Data processing

This Appendix explains the Median Absolute Deviation (MAD) criterion for data cleaning. A first step is detecting the group of leaf dry weight replicates that associate to a given leaf area. The symbol *G*(*a*) stands for this group, and the number of replicates it contains denoted by means of the symbol *n*(*G*). Formally, G(*a*) = {*w*_*i*_(*a*) | 1 ≤ *i* ≤ *n*(*G*) }. The median of *G*(*a*) is denoted by means of MED(*G*(*a*)), that is,

9 $$ \mathrm{MED}\left(G(a)\right)=\mathrm{MED}\left\{{w}_1(a),\dots .,{w}_{n(G)}(a)\right\}. $$

And, for 1 ≤ *i* ≤ *n*(*G*) the absolute deviation of replicate *w*_*i*_(*a*) from the median MED(*G*(*a*)) represented by means of *δ*_*i*_(*a*), that is,

10 $$ {\delta}_i(a)=\left|{w}_i(a)-\mathrm{MED}\left(G(a)\right)\right|. $$

Similarly, the median of the set of absolute deviations is signified by the symbol MED(*δG*(*a*)), that is,

11 $$ \mathrm{MED}\left(\delta G(a)\right)=\mathrm{MED}\left\{{\delta}_1(a),\dots .,{\delta}_{n(G)}(a)\right\}. $$

We use the character MAD(*G*(*a*)) to denote median absolute deviation of a group *G*(*a*). After Huber [47] and recalling that eelgrass leaf dry weight values are log-normally distributed, this is calculated through,

12 $$ \mathrm{MAD}\left(G(a)\right)=b\mathrm{MED}\left\{\ {\delta}_1(a),\dots .,{\delta}_{n(G)}(a)\right\}, $$

where *b* = 1/*Q*(0.75), being *Q*(0.75) the 0.75 quantile of the lognormal distribution.

The removal of inconsistent replicates in a group *G*(*a*) follows the decision criterion

13 $$ \mathrm{MED}\left(G(a)\right)-T\cdot \mathrm{MAD}\left(G(a)\right)<{w}_i(a)<\mathrm{MED}\left(G(a)\right)+T\cdot \mathrm{MAD}\left(G(a)\right), $$

where *T* is the rejection threshold. Following Miller [48], we set *T* = 3. In what follows, the symbol *n*_*qa*_ stands for the size of the resulting quality controlled data set.

## Appendix 3

### Sensitivity of *w*_*m*_(*α*, *β*, *t*)

This Appendix presents a study of the influences of parameter fluctuations in the precision of the *w*_*m*_(*α*, *β*, *t*) projections. The examination is performed on the basis of both analytical and simulation approaches. Presentation requires a formal description of variables of interest. For that aim we label a generic eelgrass shoot by using a subscript *s*. Correspondingly, the characters *nl*(*s*) and *w*_*s*_(*t*) shall stand for the associated number of leaves and their combined dry weight. Similarly, *ns*(*t*) denotes the number of shoots collected at a sampling date *t*. And, the number of sampling date symbolized by means of *n*(*t*).

Observed dry weight in a shoot *s* is represented by mean of

14 $$ {w}_s(t)={\sum}_{nl(s)}w(t), $$

where ∑_*nl*(*s*)_ indicates summation of the leaves that the shoot *s*, holds. Meanwhile, the matching average leaf dry weight of shoots denoted by the symbol *w*_*m*_(*t*) is given by

15 $$ {w}_m(t)=\frac{\sum_{ns(t)}{w}_s(t)}{ns(t)\kern0.5em }, $$

where ∑_*ns*(*t*)_ indicates summation of the *ns*(*t*) shoots collected at a time *t*.

The character 〈*w*_*m*_(*t*)〉 stands for the average of *w*_*m*_(*t*) values over the number *n*(*t*) of sampling times. It is given by

16 $$ \left\langle {w}_m(t)\right\rangle =\frac{\sum_{n(t)\kern0.75em }{w}_m(t)}{n(t)}, $$

where $\sum \limits_{n(t)}$ indicates summation over the number of sampling times.

We assumed that *w*(*t*) and *a*(*t*) are linked through the scaling expression of Eq. (1). Then, we can obtain an allometric proxy for *w*_*s*_(*t*). The symbol *w*_*s*_(*α*, *β*, *t*) represents this surrogate and it is given by

17 $$ {w}_s\left(\alpha, \beta, t\right)={\sum}_{nl(s)}\beta a{(t)}^{\alpha }. $$

Moreover,

18 $$ {w}_s(t)={w}_s\left(\alpha, \beta, t\right)+{\epsilon}_s\left(\alpha, \beta, t\right), $$

that is, *w*_*s*_(*α*, *β*, *t*) is an approximation to the true value of *w*_*s*_(*t*), being *ϵ*_*s*_(*α*, *β*, *t*) the error of estimation. Similarly, Eq. (17) produces an allometric proxy for *w*_*m*_(*t*), the average leaf dry weight in shoots at a time *t*. This surrogate, represented here by means of *w*_*m*_(*α*, *β*, *t*), is given by

19 $$ {w}_m\left(\alpha, \beta, t\right)=\frac{\sum_{ns(t)\kern0.5em }\ {w}_s\left(\alpha, \beta, t\right)}{ns(t)\kern0.5em }. $$

In turn, the average of *w*_*m*_(*α*, *β*, *t*) over the number *n*(*t*) of sampling times, denoted through 〈*w*_*m*_(*α*, *β*, *t*) 〉, is calculated by means of

20 $$ \left\langle {w}_m\left(\alpha, \beta, t\right)\ \right\rangle =\frac{\sum_{n(t)}\ {w}_m\left(\alpha, \beta, t\right)}{n(t)\ }. $$

Similarly,

21 $$ {w}_m(t)={w}_m\left(\alpha, \beta, t\right)+{\epsilon}_m\left(\alpha, \beta, t\right), $$

where *ϵ*_*m*_(*α*, *β*, *t*) stands for the resulting approximating error. Moreover, the root mean squared deviation between *w*_*m*_(*t*) and *w*_*m*_(*α*, *β*, *t*) is denoted by means of *rms*(*α*, *β*) and given by

22 $$ rms\left(\alpha, \beta \right)=\sqrt{\sum_{n(t)}{\epsilon}_m{\left(\alpha, \beta, t\right)}^2/n(t)}, $$

again ∑_*n*(*t*)_ indicates summation over the sampling dates *n*(*t*).

We assume changes in parameters *α* and *β* given by factors of their standard errors. Then, a factor *q* associates to a shift of the value of the parameter *α*. This deviation, denoted by means of the symbol *α*_*q*_, is given by

23 $$ {\alpha}_q=\alpha +\varDelta {\alpha}_q, $$

where

24 $$ {\Delta \alpha}_q=q\cdot ste\left(\alpha \right), $$

with *ste* (*α*) symbolizing the standard error of *α* and *q* satisfying.

25 $$ \mid q\mid \le {q}_{\max .} $$

Similarly, a factor *r*, such that.

26 $$ \left|r\right|\le {r}_{\mathit{\max},} $$

associates to a fluctuation in *β* denoted by means of *Δβ*_*r*_ and expressed by way of

27 $$ \varDelta {\beta}_r=r\cdot ste\left(\beta \right), $$

being *ste*(*β*) the standard error of *β*. This returns a variation of the parameter *β*, denoted via *β*_*r*_ and represented in the form

28 $$ {\beta}_r=\beta +\varDelta {\beta}_r. $$

### Qualitative exploration of sensitivity of ***w***_***m***_(***α***, ***β***, ***t***)

A qualitative study is intended to set domains where reference estimations *w*_*m*_(*α*, *β*, *t*) are underestimated or overestimated by changing values *w*_*m*_(*α*_*q*_, *β*_*r*_, *t*).For that aim, we firstly set reasonable local variation ranges for the numerical deviations *α*_*q*_ and *β*_*r*_. Fittings of Eq. (1) to eelgrass leaf dry weight and area data demonstrate that the inequality 0 < *β* < *α* holds [30]. As stated by Eqs. (24) and (27), letting |*q*| < *ste*(*α*)/*α* along with | *r*| < *ste*(*β*)/*β* set domains |*∆α*_*q*_| ≤ *α* and |*∆β*_*r*_| ≤ *β*. This enables suitable domain to pursue a qualitative study of the effects of parametric changes in *w*_*s*_(*α*, *β*, *t*) (cf. Eq. (17)). For that aim, let’s consider deviations *δw*_*s*_(*α*_*q*_, *β*_*r*_, *t*) defined through

29 $$ \delta {w}_s\left({\alpha}_q,{\beta}_r,t\right)={w}_s\left({\alpha}_q,{\beta}_r,t\right)-{w}_s\left(\alpha, \beta, t\right). $$

Then,

30 $$ \delta {w}_s\left({\alpha}_q,{\beta}_r,t\right)={\sum}_{nl(s)}\left(\left(\beta +\varDelta {\beta}_r\right)a{(t)}^{\alpha +\varDelta {\alpha}_q}-\beta a{(t)}^{\alpha}\right), $$

and factoring the term *βa*(*t*)^*α*^

31 $$ \delta {w}_s\left({\alpha}_q,{\beta}_r,t\right)={\sum}_{nl(s)}\beta a{(t)}^{\alpha }{\mu}_a\left(\varDelta {\alpha}_q\kern0.5em \varDelta {\beta}_r,a(t)\right), $$

where

32 $$ {\mu}_a\left(\varDelta {\alpha}_q\kern0.5em \varDelta {\beta}_r,a(t)\right)=\frac{\pi \left(\varDelta {\alpha}_q\right)}{\ \varphi \left(\varDelta {\beta}_r\right)}-1 $$

with

33 $$ \varphi \left(\varDelta {\beta}_r\right)=\frac{\beta }{\beta +\varDelta {\beta}_r} $$

and

34 $$ \pi \left(\varDelta {\alpha}_q\right)=a{(t)}^{\varDelta {\alpha}_q}. $$

Now, since *a*(*t*) is positive, for fixed values of the parameters *α* and *β* in the domain |*∆α*_*q*_| < *α*, the factor *π*(*∆α*_*q*_) remains positive and increasing. Respectively, for *∆β*_*r*_ varying in the interval |*∆β*_*r*_| < *β*, the factor *φ*(*∆β*_*r*_) is positive and decreasing. Furthermore, for *∆α*_*q*_ = *∆β*_*r*_= 0, we have *π*(*∆α*_*q*_) = *φ*(*∆β*_*r*_) = 1 (Fig. 16).

From Eq. (31) the proxies *w*_*s*_(*α*, *β*, *t*) will be overestimated by shifting projections *w*_*s*_(*α*_*q*_, *β*_*r*_, *t*) whenever the inequality

35 $$ {\mu}_a\left(\varDelta {\alpha}_q,\varDelta {\beta}_r,a(t)\right)\kern0.5em >0 $$

is satisfied. And from Eq. (32) this last inequality leads to

36 $$ \pi \left(\varDelta {\alpha}_q\right)>\varphi \left(\varDelta {\beta}_r\right). $$

But, for −*α* ≤ *∆α*_*q*_ ≤ 0 the factor *π*(*∆α*_*q*_) is continuous and increases monotonically from a value *π*(−*α*) > 0, until it reaches a value of *π*(0) = 1, while the factor *φ*(*∆β*_*r*_) takes positive and arbitrarily large values whenever *∆β*_*r*_ approaches –*β*, and decreases monotonically towards a value of *φ*(0) = 1. Thus, the continuity of *φ*(*∆β*_*r*_) and *π*(*∆α*_*q*_) implies the order relationship *π*(*∆α*_*q*_) < *φ*(*∆β*_*r*_), in the domain−*α* ≤ *∆α*_*q*_ ≤ 0 and –*β* < *∆β*_*r*_ ≤ 0. In the other hand in the domain 0 ≤ *∆α*_*q*_ ≤ *α* and 0 ≤ *∆β*_*r*_ ≤ *β*, both *π*(*∆α*_*q*_) and *φ*(*∆β*_*r*_) steer away from one being *π*(*∆α*_*q*_) increasing and *φ*(*∆β*_*r*_) decreasing. Therefore, by continuity the statement of inequality (36) will hold only in the domain *∆α*_*q*_ > 0 and *∆β*_*r*_ > 0.

Similarly, reference values *w*_*s*_(*α*, *β*, *t*) will be underestimated by fluctuating ones *w*_*s*_(*α*_*q*_, *β*_*r*_, *t*) whenever, the statement

37 $$ {\mu}_a\left(\varDelta {\alpha}_q,\varDelta {\beta}_r,a(t)\right)<0 $$

holds, or equivalently whenever

38 $$ \pi \left(\varDelta {\alpha}_q\right)<\varphi \left(\varDelta {\beta}_r\right), $$

which as it has been discussed above, only holds whenever the increments *∆α*_*q*_ and *∆β*_*r*_ vary in.

the domain −*β* < *∆β*_*r*_ < 0 and −*α* < *∆α*_*q*_ < 0.

Since, the *w*_*s*_(*α*, *β*, *t*) projections are always positive, the *w*_*m*_(*α*, *β*, *t*) reference projections will be overestimated or underestimated by changing *w*_*m*_(*α*_*q*_, *β*_*r*_, *t*) values in the same domains of variation of *∆α*_*q*_ and and *∆β*_*r*_ where the *w*_*s*_(*α*, *β*, *t*) projections are overestimated or underestimated by *w*_*s*_(*α*_*q*_, *β*_*r*_, *t*) values.

### Exploration of sensitivity of *w*_*m*_(*α*, *β*, *t*) by simulation methods

In order to perform a simulation study of sensitivity, we considered a combined range of variability for *α* and *β* given by the modulus of the vector of parametric changes (*∆α*_*q*_, *∆β*_*r*_). This is denoted by means of the symbol *θ*(*q*, *r*) and calculated through

39 $$ \theta \left(q,r\right)=\sqrt{\ \varDelta {\alpha_q}^2+\varDelta {\beta_r}^2.} $$

Equivalently, using Eqs. (24) and (27) yield

40 $$ \theta \left(q,r\right)=\sqrt{{\left(q\cdot ste\left(\alpha \right)\right)}^2+{\left(r\cdot ste\left(\beta \right)\right)}^2.} $$

And, setting *q* = 1 and *r* = 1 give (*∆α*_*q*_, *∆β*_*r*_) = (*ste*(*α*), *ste*(*β*)). This produces vectors of shifting parametric values with modulus determined by the standard errors of estimates. Denoting by means of the symbol *θ*_*ste*_ the associated range of variability we have

41 $$ {\theta}_{ste}=\sqrt{ste{\left(\alpha \right)}^2+ ste{\left(\beta \right)}^2}. $$

By virtue of Eq. (39), every fixed value of *θ*(*q*, *r*) returns different pairs (*∆α*_*q*_, *∆β*_*r*_) of increments, each one giving rise to a couple of shifting parameter values (*α*_*q*_, *β*_*r*_). Besides, for each ordered pair of changing parameters, leaf data in shoots and Eq. (19) return a shifting trajectory, that is denoted trough the symbol *w*_*m*_(*α*_*q*_, *β*_*r*_, *t*). The average of the values taken by the fluctuating trajectories at a sampling date *t* is denoted by 〈*w*_*m*_(*α*_*q*_, *β*_*r*_, *t*)| *θ*〉. The procedure estimates the deviations between the observed *w*_*m*_(*t*) and the average 〈*w*_*m*_(*α*_*q*_, *β*_*r*_, *t*)| *θ*〉 trajectory for each time *t*. This is denoted by *δw*_*mθ*_(*α*_*q*_, *β*_*r*_, *t*) and calculated by way of

42 $$ \delta {w}_{m\theta}\left({\alpha}_q,{\beta}_r,t\right)={w}_m(t)-\left\langle {w}_{m\kern0.5em }\left({\alpha}_q,{\beta}_r,t\right)|\theta \right\rangle . $$

The average of the *δw*_*mθ*_(*α*_*q*_, *β*_*r*_, *t*) deviation values over all sampling times *n*(*t*), represented thorough the symbol 〈*δw*_*mθ*_(*α*_*q*_, *β*_*r*_, *t*)〉, is given by

43 $$ \left\langle \delta {w}_{m\theta}\left({\alpha}_q,{\beta}_r,t\right)\right\rangle =\frac{\sum_{n(t)}\ \delta {w}_{m\theta}\left({\alpha}_q,{\beta}_r,t\right)\kern0.5em }{n(t)}, $$

where, $\sum \limits_{n(t)}$ indicates summation over the involved *n*(*t*) sampling dates. These statistics along with, the average of *w*_*m*_(*t*) values over the number *n*(*t*) of sampling times are used to produce a relative deviation index represented by means of the symbol *ϑ*(*θ*) and given by

44 $$ \vartheta \left(\theta\ \right)=\frac{\left|\left\langle \delta {w}_{m\theta}\left({\alpha}_q,{\beta}_r,t\right)\right\rangle \right|\kern0.5em }{\left\langle {w}_m(t)\right\rangle\ }. $$

The value of *ϑ*(*θ*) stands a measure of the sensitivity of the reference trajectory *w*_*m*_(*α*, *β*, *t*) to a change of tolerance *θ*(*q*, *r*) on the values of *α* and *β*. Moreover, *ϑ*(*θ*) assess in what percentage of 〈*w*_*m*_(*t*)〉 the absolute deviation between *w*_*m*_(*α*_*q*_, *β*_*r*_, *t*) and *w*_*m*_(*t*) amounts.

### Application to present data

The results of Appendix 1 show that there are not differences in parameter estimates of the nonlinear regression model of Eqs. (5) and (6) assuming heteroscedasticity or homoscedasticity. Therefore, in order to explore the sensitivity of *w*_*m*_(*α*, *β*, *t*) by means of the simulation code of Eqs. (39)–(44), we take as a reference parameter estimates acquired from the raw data using the homoscedastic case. Parameter estimates were $\widehat{\alpha}=1.104$ with $ste\left(\widehat{\alpha}\ \right)=5.101\times {10}^{-3}$ for *α* and of $\widehat{\beta}=8.71\times {10}^{-6}$ with $ste\left(\widehat{\beta}\right)=3.53\times {10}^{-7}$ for *β* (Table 1). Then, letting *q* = 1 and *r* = 1 in Eqs. (24) and (27) allows to consider a combined range of parametric change set by the standard errors of estimates. Eq. (41) returned *θ*_*ste*_ = 5.101 × 10^−3^. Afterwards, for every fixed value of *θ*(*q*, *r*) we determined all ordered pairs (*∆α*_*q*_, *∆β*_*r*_) complying with condition set by Eq. (39). This returned fluctuating values *α*_*q*_ for *α* and *β*_*r*_ for *β*, given one by one by Eqs. (23) and (28). Observed leaf area data and shifting parameter values, shaped the associated changing *w*_*m*_(*α*_*q*_, *β*_*r*_, *t*) trajectories. Moreover, while 0 ≤ *θ*(*q*, *r*) ≤ *θ*_*ste*_, the values of the relative deviation index *ϑ*(*τ*) satisfied 0≤ *ϑ*(*θ*) ≤ 0.0205 (Fig. 9). Thus, for a parametric variation range set by the standard errors of estimates the maximum value of the absolute deviation *ϑ*(*θ*) between *w*_*m*_(*α*_*q*_, *β*_*r*_, *t*) and *w*_*m*_(*t*) amounts to approximately 2% of the average of *w*_*m*_(*t*) taken over all sampling dates (〈*w*_*m*_(*t*) 〉 cf. Eq. (16)). Moreover, for *θ*(*q*, *r*) in a range 0 ≤ *θ* ≤ 2*θ*_*ste*_ we have 0 ≤ *ϑ*(*θ*) ≤ 0.031. This leads to a maximum absolute deviation between *w*_*m*_(*α*_*q*_, *β*_*r*_, *t*) and *w*_*m*_(*t*) of around 3% of 〈*w*_*m*_(*t*) 〉. This shows the robustness of the *w*_*m*_(*α*, *β*, *t*) projection method.

In turn, fitting the model of Eq. (1) to the quality controlled data using nonlinear regression, produced estimates, $\widehat{\alpha}=1.132$ with $ste\left(\widehat{\alpha}\ \right)=3.954\times {10}^{-3}$ for *α* and of $\widehat{\beta}=6.956\times {10}^{-6}$ with$ste\left(\hat{\beta}\right)=2.202\times {10}^{-7}$ for *β* (Table 1). Letting *p* = 1 and *q* = 1 in Eq. (40) gives *θ*_*ste*_ = 3.954 × 10^−3^, therefore, 0 ≤ *θ*(*q*, *r*) ≤ *θ*_*ste*_ implies 0 ≤ *ϑ*(*θ*) ≤ 0.003 (Fig. 10). Hence, the maximum absolute-relative deviation between *w*_*m*_(*α*_*q*_, *β*_*r*_, *t*) and *w*_*m*_(*t*) amounts to only 0.03% of the 〈*w*_*m*_(*t*)〉 average. On addition, a range 0 ≤ *θ* ≤ 2.8*θ*_*ste*_ leads to 0 ≤ *ϑ*(*θ*)  ≤ 0.03006. This leads to a maximum absolute deviation between *w*_*m*_(*α*_*q*_, *β*_*r*_, *t*) and *w*_*m*_(*t*) of around 3% of 〈*w*_*m*_(*t*)〉. Comparing with results for raw data, we can ascertain that data quality induces a relative gain in the precision of the *w*_*m*_(*α*, *β*, *t*) projections.
